# Dysregulated affective arousal regulates reward-based decision making in patients with schizophrenia: an integrated study

**DOI:** 10.1038/s41537-022-00234-y

**Published:** 2022-03-21

**Authors:** Hong-Hsiang Liu, Chih-Min Liu, Ming H. Hsieh, Yi-Ling Chien, Yung-Fong Hsu, Wen-Sung Lai

**Affiliations:** 1 Department of Psychology, National Taiwan University, Taipei, Taiwan; 2 Department of Psychiatry, National Taiwan University Hospital, Taipei, Taiwan; 3 Graduate Institute of Brain and Mind Sciences, National Taiwan University, Taipei, Taiwan; 4 Neurobiology and Cognitive Science Center, National Taiwan University, Taipei, Taiwan

**Keywords:** Human behaviour, Schizophrenia

## Abstract

Schizophrenia is a chronic and severe mental disorder. Dysregulated decision-making and affective processing have been implicated in patients with schizophrenia (SZ) and have significant impacts on their cognitive and social functions. However, little is known about how affective arousal influences reward-based decision-making in SZ. Taking advantage of a two-choice probabilistic gambling task and utilizing three facial expressions as affective primes (i.e., neutral, angry, and happy conditions) in each trial, we investigated how affective arousal influences reward-related choice based on behavioral, model fitting, and feedback-related negativity (FRN) data in 38 SZ and 26 healthy controls (CTRL). We also correlated our measurements with patients’ symptom severity. Compared with the CTRL group, SZ expressed blunted responses to angry facial primes. They had lower total game scores and displayed more maladaptive choice strategies (i.e., less win-stay and more lose-shift) and errors in monitoring rewards. Model fitting results revealed that the SZ group had a higher learning rate and lower choice consistency, especially in the happy condition. Brain activity data further indicated that SZ had smaller amplitudes of FRN than their controls in the angry and happy conditions. Importantly, the SZ group exhibited attenuated affective influence on decision-making, and their impairments in decision-making were only correlated with their clinical symptoms in the angry condition. Our findings imply the affective processing is dysregulated in SZ and it is selectively involved in the regulation of choice strategies, choice behaviors, and FRN in SZ, which lead to impairments in reward-related decision-making, especially in the angry condition.

## Introduction

Schizophrenia is a severe mental illness that affects approximately 1% of the world’s population. Aberrant midbrain dopamine level has been proposed as one of the key features in schizophrenia^1^. Mounting evidence has linked it to dysregulated decision making, as the updated reward prediction error (RPE)—a discrepancy between the predicted and actual action outcomes—is encoded by dopaminergic systems^2,3^. Abnormalities in the dopamine system have been considered to alter the appraisal of stimuli and reward processing in patients with schizophrenia (SZ)^4,5^. These findings have now led to the neuroeconomic approach of schizophrenia research, which brings out a broad set of tools and experimental paradigms to characterize altered reward processing and choice behavior in psychiatric populations^5,6^. For instance, Juckel et al.^7^ applied the monetary incentive delay task to unmedicated SZ. They found that, compared with healthy controls (CTRLs), SZ had reduced activation in ventral striatal dopamine projections during reward anticipation. In a reward-related prediction-error task, Morris et al.^8^ reported that the differential activation between expected and unexpected feedbacks was attenuated in SZ, which was due to exaggerated ventral striatal responses to expected rewards and blunted responses to unexpected outcomes. The results of these studies have been interpreted as dysregulated reward processing and RPE signaling in SZ. Furthermore, Li, Lai, Liu, and Hsu^9^ examined the mechanisms related to this dysregulated reward processing through model fitting. They found that SZ exhibited lower reward sensitivity with more frequently updated reward representation than CTRL in a probabilistic gambling task. Importantly, they found that patients’ unstable choice behavior appeared to be correlated with their scores on P1 “delusion” and P3 “hallucinatory behavior” in the Positive and Negative Syndrome Scale (PANSS)^10^, suggesting a link between dysregulated decision making and the severity of psychotic symptoms.

In addition to the associative-based reinforcement learning model, the altered reward-based decision making in SZ was also investigated with rule-based win-stay/lose-shift strategies^11,12^. From an operant learning view, a rewarded choice should be repeated (i.e., win-stay), whereas the choice leads to negative outcome should subsequently be avoided in the next round (i.e., lose-shift)^13,14^. In a probabilistic reversal learning task, it was reported that SZ has a trend level of less frequent win-stay and more frequent lose-shift than CTRL^11^. With a similar design, Waltz et al.^12^ also found that the patients exhibited significantly more frequent shifts to rich option (i.e., high reward probability) than CTRL regardless of the feedback type. Thus, these studies suggest that the robustness of patients’ internal representation of correct choice might be decreased, which render them to have a reduced propensity for adaptive win-stay/lose-shift strategies.

Along with dysregulated decision making, SZ also suffer from affective disturbances. Existing literature suggests that patients have significantly worse performance in affect perception than CTRLs across a range of sensory modalities^15^, and this abnormality appears to be more specific to the interpretation of negative affect than to positive ones^16–19^. For instance, Horley et al.^17^ analyzed participants’ electroencephalograms (EEGs) when they were passively viewing facial pictures and found that SZ displayed a delayed and diminished frontal P200 response to angry faces compared with CTRLs. The P200 component has been reported to reflect the initial stages of facial expression processing and emotion analysis^20–25^. Therefore, their finding suggests a blunted affective response to angry faces in SZ. Intriguingly, compared to CTRLs, Premkumar et al.^18^ reported that SZ not only had lower accuracy in recognizing negative affect but also exhibited significant misattribution bias—they tended to misattribute angry to neutral expressions. Thus, the blunted and attenuated response to negative affect might be prominent features of affective disturbance in SZ.

Given that dysregulated decision-making and affective processing have both been implicated in SZ and have significant impacts on their cognitive and social functions, it is of interest to investigate how these two deficits interact in this population. In this study, we took advantage of facial expression primes (i.e., neutral, happy, and angry faces) from a culture-based facial expression database to elicit affective responses and measured choice behaviors in a probabilistic gambling task as reported previously^26^. In addition to conventional behavioral data, an integrated approach from choice strategy, model-fitting, and EEG perspectives was conducted to characterize the deficits in reward-based decision making and to determine the mental processes that underlie choice behavior. In particular, we analyzed the win-stay/lose-shift strategies in each participant to untangle their choice behavior^11–14^, adopted a simplified reinforcement learning model to unfold the mental processes underlying choice behavior, and applied an EEG component termed feedback-related negativity (FRN, a neural index of prediction error)^27–29^ to index RPE signaling that driving reward-based decision making^30–33^. Our measurements across three prime conditions were also correlated with symptom subscores of the PANSS to reveal the correlation between each index and symptom severity in these patients.

## Results

### Significant interaction between group and prime condition in su**b**jective affective response was identified

**Fig. 1:**
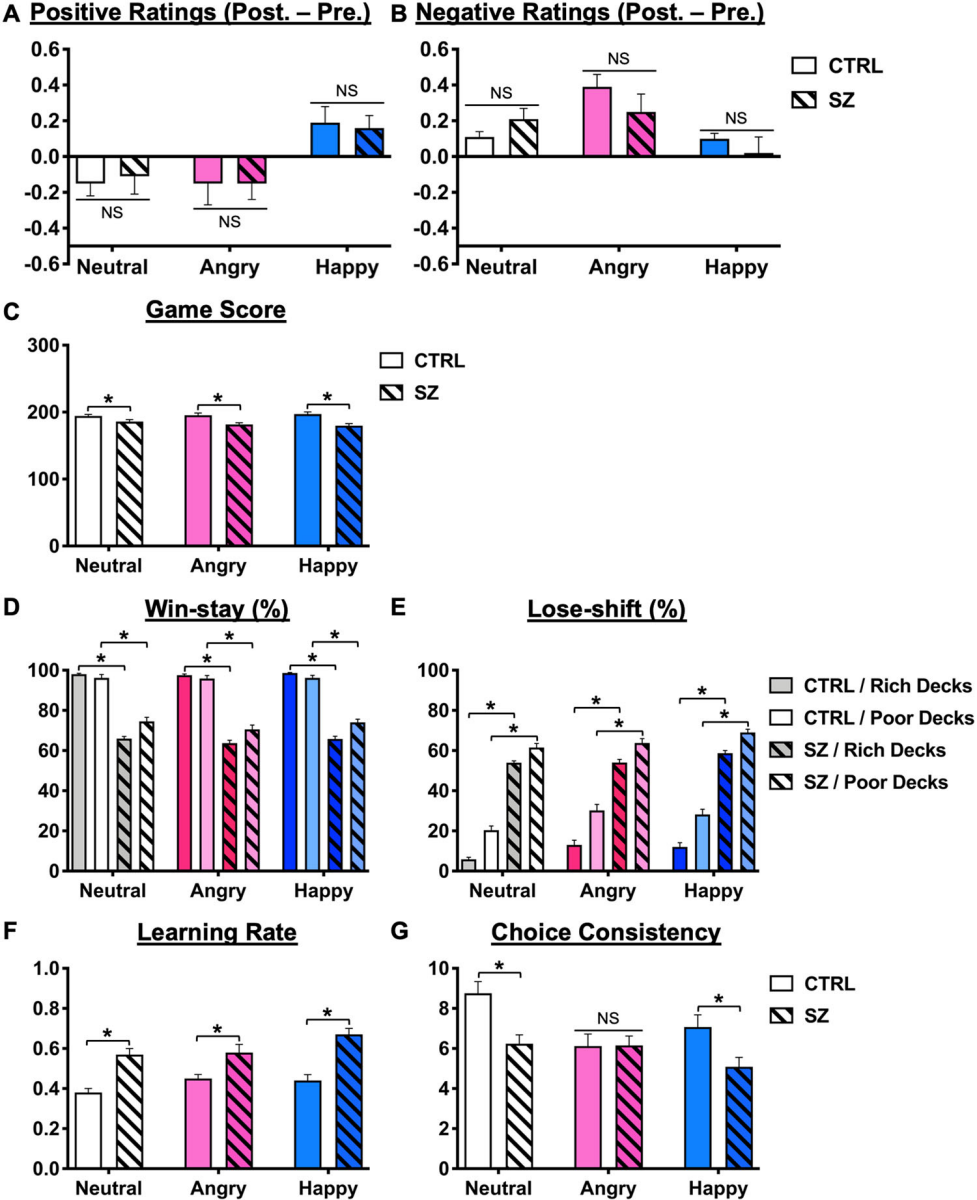
Behavioral and model-fitting results. **A**, **B** Difference between pretest and posttest mean (±SEM) rating scores of the positive-affect and negative-affect subscales from PANAS, respectively. Mean difference scores of the healthy controls (CTRL, open bars) and patients with schizophrenia (SZ, striped bars) groups in the neutral (bars in white/gray), angry (bars in pink/light pink), and happy (bars in blue/light blue) facial prime conditions are shown. **C** Mean (±SEM) game scores. **D**, **E** Mean (±SEM) percentage of win-stay and lose-shift. The results of rich and poor decks are illustrated in black and gray lines, respectively. **F**, **G** Mean (±SEM) learning rate and choice consistency, respectively. *p < 0.05 between CTRL and SZ groups in the facial prime condition.

For subjective ratings of affective responses, there was a significant group × prime condition × PANAS item interaction [*F*(2,124) = 12.23, *p* < 0.001]. For positive ratings (Fig. 1A), both the CTRL and SZ groups had higher ratings in the happy conditions than in the neutral and angry conditions (all *p* < 0.05). However, no significant difference was found between the two groups in each condition (all *p* > 0.05). For negative ratings (Fig. 1B), the CTRL group had a significantly higher rating in the angry condition than in the other two conditions (both *p* < 0.05), whereas no significant difference between conditions was found in the SZ group (all *p* > 0.05).

### SZ had worse task performances and displayed different choice strategies compared to CTRL

As indicated in Fig. 1C, the SZ group had lower total game scores than the CTRL group in all 3 facial prime conditions (all *p* < 0.05). Their choice behavior was further revealed by analyzing win-stay/lose-shift strategies. For the win-stay strategy (Fig. 1D), there were significant main effects for group [*F*(1,62) = 711.89, *p* < 0.001] and deck [*F*(1,62) = 7.40, *p* < 0.01] and their interaction [*F*(1,62) = 20.58, *p* < 0.001]. Post hoc analyzes revealed that the SZ group exhibited an overall lower percentage of win-stay (69.07% ± 10.91%) than the CTRL group (97.09% ± 5.64%) across three conditions. The CTRL group overall exhibited more win stays in the choice of the rich deck than the poor decks (*p* < 0.01), whereas the SZ group overall displayed the opposite pattern (rich deck < poor deck, *p* < 0.01). For the lose-shift strategy (Fig. 1E), there were significant main effects for group [*F*(1,62) = 606.46, *p* < 0.001], deck [*F*(1,62) = 173.79, *p* < 0.001], prime condition [*F*(2,124) = 13.11, *p* < 0.001], and their interaction [*F*(2,124) = 4.414, *p* < 0.05]. Post hoc analyzes revealed that the SZ group overall exhibited a higher percentage of lose-shift (60.19% ± 11.28%) than the CTRL group (18.28% ± 14.43%) across the three conditions. Specifically, the CTRL group overall exhibited higher percentages of lose-shift in angry and happy conditions than in neutral condition (all *p* < 0.01), whereas SZ only had a higher percentage of lose-shift in the happy condition compared to neutral and angry conditions (all *p* < 0.01), but no difference was found between neutral and angry conditions. Together, the win-stay/lose-shift results suggest that angry faces had less impact on choice strategy in the SZ group and that they were unable to optimize their action in the task.

### Model-based analysis revealed an altered learning rate and choice consistency in SZ

For the learning rate (*α*), there were significant main effects for group [*F*(1,62) = 21.93, *p* < 0.01], prime condition [*F*(2,124) = 7.73, *p* < 0.01], and their interaction [*F*(2,124) = 3.61, *p* < 0.05], as depicted in Fig. 1F. Post hoc analyzes revealed that the SZ group overall had a higher learning rate than the CTRL group across three conditions, suggesting an unstable and rapidly updated value representation in SZ. Compared to the neutral condition, the CTRL group had higher learning rates in both emotional conditions, whereas the SZ group only had a higher learning rate in the happy condition (all *p* < 0.05).

For choice consistency (*β*), there were significant main effects for group [*F*(1,62) = 6.25, *p* < 0.01], prime condition [*F*(2,124) = 8.82, *p* < 0.01], and their interaction [*F*(2,124) = 6.18, *p* < 0.01] (Fig. 1G). Post hoc analyzes revealed that the SZ group overall had a lower choice consistency than the CTRL group, suggesting that SZ had unstable and exploratory choice behaviors. The CTRL group had a higher choice consistency in the neutral condition than in the angry and happy conditions, whereas the SZ group had a lower choice consistency in the happy condition than in the neutral and angry conditions (all *p* < 0.05).

### Alterations of structural encoding of facial features, objective affective responses, and FRNs were revealed in SZ

**Fig. 2:**
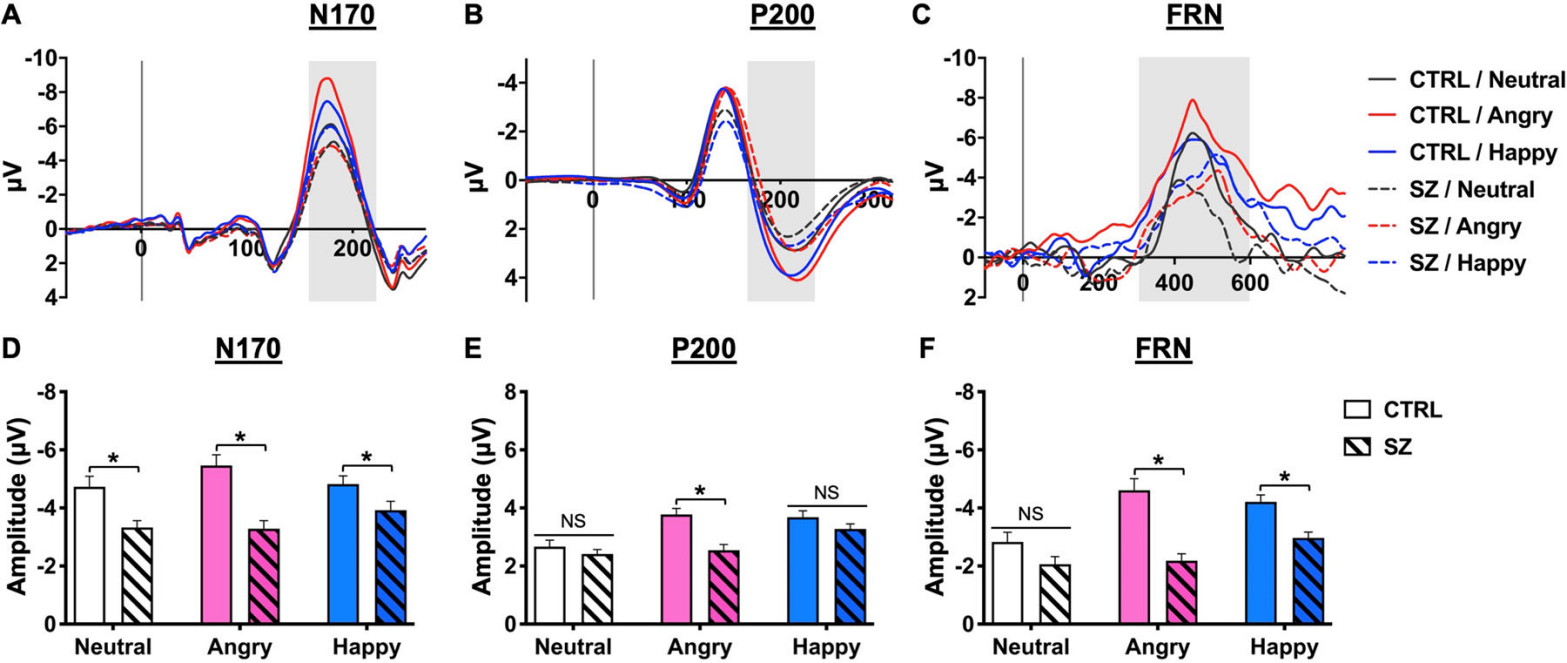
EEG results. **A**–**C** Grand averaged waveforms of the N170, P200, and FRN for the control (CTRL, solid lines) and patients with schizophrenia (SZ, dashed lines) groups in the neutral (gray lines), angry (red lines), and happy (blue lines) conditions. **D**–**F** Mean (±SEM) amplitudes of the N170, P200, and FRN for the healthy controls (CTRL, open bars) and patients with schizophrenia (SZ, striped bars) groups in the neutral (white bars), angry (pink bars), and happy (blue bars) prime conditions. *p < 0.05 between CTRL and SZ groups in the facial prime condition.

The N170 ERP component has been widely identified as a face-sensitive neural marker. The grand averages of the N170 waveforms at the P8 channel and the corresponding mean amplitudes are shown in Fig. 2A, D, respectively. There were pronounced main effects for the electrode [*F*(1,62) = 34.22, *p* < 0.001] and group [*F*(1,62) = 332.07, *p* < 0.001]. Post hoc analyzes revealed that N170 was significantly right lateralized (P7: −3.7 ± 0.16 μV; P8: −4.54 ± 0.20 μV), as reported previously^34,35^. Importantly, the overall mean amplitude of the N170 in SZ group (−3.51 ± 0.16 μV) was smaller than the one in the CTRL group (−5.01 ± 0.20 μV). There were significant group differences between SZ and CTRL across three facial prime conditions (Fig. 2D, all *p* < 0.05). Compared to CTRL, the N170 result suggests a disrupted structural encoding of facial features in SZ.

As depicted in Fig. 2B, E, objective affective responses were characterized by the grand averages of the P200 waveforms at the FCz channel and the corresponding mean amplitudes. There were significant effects for the prime condition [*F*(2,124) = 10.92, *p* < 0.001] and its interaction with the group [*F*(2,124) = 3.44, *p* < 0.05]. Post hoc analyzes revealed that the CTRL group exhibited a larger P200 in the angry and happy conditions than in the neutral condition (both *p* < 0.001), suggesting a higher level of affective response to emotional faces. In contrast, the SZ group had a significantly larger P200 in the happy condition than in the other two conditions (both *p* < 0.05), but no difference was found between the angry and neutral conditions. Importantly, a simple main effect between SZ and CTRL was found only in angry condition (Fig. 2E). Complementary to the subjective ratings, these findings indicated that SZ had an attenuated affective response to angry faces.

For RPE signaling, the grand averages of FRN waveforms at the FCz channel and the corresponding mean amplitudes are shown in Fig. 2C, F. There were significant main effects for group [*F*(1,62) = 26.57, *p* < 0.01], prime condition [*F*(2,124) = 9.08, *p* < 0.01], and their interaction [*F*(2,124) = 4.43, *p* < 0.05]. Post hoc analyzes revealed that the SZ group overall had a smaller amplitude of FRN than the CTRL group (−2.40 ± 0.18 vs. −3.88 ± 0.22 μV). Compared to the neutral condition, the CTRL group had larger FRN in both angry and happy conditions, whereas the SZ group only exhibited a larger FRN in the happy condition (all *p* < 0.01, Fig. 2F). Importantly, compared to the CTRL group, the SZ group only exhibited significant group differences in the two emotional conditions, especially in the angry condition (Fig. 2F, both *p* < 0.05). This result suggests that SZ not only had aberrant RPE but also had an attenuated affective influence on reward processing, especially in angry and happy conditions.

### The correlations between task measurements and the five factors of PANSS

The correlations between the five factors of the PANSS and our measurements across three emotional conditions in the SZ group are indicated in Table 1. For the structural encoding of facial features, no significant association was found between the amplitude of N170 and any symptom subscore. In contrast, for affective response, there were significantly negative correlations between P200 amplitude and the negative and cognitive factors of PANSS in the angry prime condition. However, none of the correlations survived after the Holm–Bonferroni correction. For model fitting parameters, the learning rate in the angry prime condition is marginally correlated with the positive factor, whereas the choice consistency in the angry prime condition is significantly negatively correlated with the positive factor. For the FRN in the angry prime condition, there were significantly negative correlations between its amplitude and both the positive and cognitive factors of PANSS. However, only the correlations between FRN in the angry prime condition and PANSS factors survived after the Holm–Bonferroni correction.

**Table 1:** Correlations between task measurements and symptom severity in patients with schizophrenia.

		Positive	Negative	Cognitive	Excitement	Depressive
P200	Pearson’s *r*	−0.273	−0.267	−0.263	0.153	0.009
(Neutral condition)	*p*-value	0.097	0.105	0.111	0.360	0.957
P200	Pearson’s *r*	−0.291	**−0.362**^a^	**−0.344**^a^	−0.133	−0.178
(Angry condition)	*p*-value	0.076	**0.025**	**0.035**	0.426	0.284
P200	Pearson’s *r*	−0.226	−0.154	−0.267	0.081	−0.109
(Happy condition)	*p*-value	0.172	0.355	0.104	0.629	0.514
Learning rate	Pearson’s *r*	0.046	−0.178	−0.056	0.130	−0.077
(Neutral condition)	*p*-value	0.786	0.286	0.738	0.436	0.645
Learning rate	Pearson’s *r*	0.310	0.113	0.015	−0.011	−0.253
(Angry condition)	*p*-value	0.058	0.498	0.930	0.947	0.125
Learning rate	Pearson’s *r*	0.197	0.078	0.127	−0.025	−0.124
(Happy condition)	*p*-value	0.235	0.642	0.449	0.884	0.456
Choice consistency	Pearson’s *r*	−0.307	0.026	−0.150	−0.017	0.164
(Neutral condition)	*p*-value	0.061	0.876	0.367	0.920	0.325
Choice consistency	Pearson’s *r*	**−0.334**^a^	−0.093	−0.181	0.066	−0.046
(Angry condition)	*p*-value	**0.040**	0.580	0.276	0.695	0.784
Choice consistency	Pearson’s *r*	−0.059	−0.254	−0.132	0.031	0.189
(Happy condition)	*p*-value	0.726	0.124	0.428	0.855	0.256
FRN	Pearson’s *r*	−0.289	−0.007	−0.091	−0.037	−0.126
(Neutral condition)	p-value	0.078	0.969	0.587	0.825	0.451
FRN	Pearson’s *r*	**−0.436**^b^	−0.023	**−0.447**^b^	−0.062	−0.207
(Angry condition)	*p*-value	**0.006**	0.891	**0.005**	0.712	0.213
FRN	Pearson’s r	−0.271	−0.079	−0.196	0.047	−0.055
(Happy condition)	*p*-value	0.099	0.638	0.239	0.781	0.741

Differences considered as statistically significant (*p* < 0.05) are marked by bold fonts.

^a^Statistically significant.

^b^Statistically significant after the Holm–Bonferroni correction.

## Discussion

In this study, we incorporated behavioral, model-fitting, and ERP measures to characterize how affective response induced by facial primes regulate reward-based decision making in a gambling task and, importantly, how this process is altered in SZ. In our CTRL group, the findings are consistent with our previous study in which both angry and happy faces evoked higher levels of affective responses than neutral faces^26^. These results imply that the presentation of facial expressions can prime affective responses, which then intensifies RPE signaling, interrupts reward expectations, and leads to exploratory choices, supporting the significant emotion regulation effect on decision making^36^. In addition, a novel and intriguing result was found regarding the affective modulation on participants’ win-stay/lose-shift strategies. Previous studies have identified that abnormal affective arousal reduces the propensity of advantageous strategies and hinders individual’s choice behavior^37,38^. For instance, Dickstein et al.^37^ compared the choice behavior of youths with bipolar disorder (BD), severe mood dysregulation (SMD), major depressive disorder (MDD), and anxiety disorder (ANX) with healthy controls in a probabilistic response reversal task. They found that BD and SMD youths have less frequent win-stay and lose-shift to rich option than controls. With a similar design, Xia, Xu, Yang, Gu, Zhang^38^ compared the choice behavior between high and low trait anxiety in college students, and found that high trait anxiety students exhibited less frequent loss-shift than those with low trait anxiety. In complementary to these findings, in the present study, we took one step further and showed that the frequency of lose-shift would be exaggerated by merely priming the participants with facial expressions even in healthy controls. Therefore, our finding suggests that the choice strategies during reward-based decision making might be more sensitive to individual’s affective state than previous reports.

In agreement with previous studies^7–9,11,12^, we identified significant impairments in reward processing. The SZ group had lower monetary rewards (i.e., total game scores) than the CTRL group across all three facial prime conditions. SZ overall exhibited dysregulated choice strategies (i.e., less win-stay and more lose-shift), impaired error monitoring (i.e., more win-stay in the choice of poor deck than rich deck), fragile value representation (i.e., increased learning rate), unstable choice consistency (i.e., low choice consistency), and disturbed RPE signaling (i.e., attenuated FRN). Intriguingly, these impairments are mainly correlated with the positive factor of PANSS. More importantly, it is of great interest to observe and reconfirm impairments of structural and affective facial processing in SZ group. Compared to CTRL group, the reduction of N170 amplitude in our SZ suggests these patients have a disturbed structural encoding of facial features in all three facial prime conditions. Even though it is not associated with any symptom subscore, it is in line with previous findings on the facial recognition deficits in schizophrenia^39,40^. Furthermore, our findings from both subjective (i.e., PANAS ratings) and objective (i.e., P200 amplitude) measurements of affective responses consistently indicated that SZ showed a lower level of affective responses to the angry faces than either the neutral or happy ones. This result is in line with the findings of an attenuated affective response^17^ and misattribution bias^18^ to negative facial expressions in previous studies. In addition, our data support that the level of disturbance has a trend of correlation (i.e., after Holm–Bonferroni sequential correction) with the severity of both negative and cognitive factors of PANSS, which is consistent with previous findings in multiepisode schizophrenia^41^.

Importantly, the perception of facial expressions is a vital mediator between cognitive and social functions. In SZ, blunted responses to emotional faces could cause them to ignore salient information (e.g., angry expression of others) during social communication, which might lead to disturbances in interpersonal skills^42,43^. In a similar vein, we reported that affective disturbance in SZ also dysregulated their reward-based decision making in this study. Compared to CTRL, our patients had a comparable level of FRN in the neutral condition. This finding is consistent with a recent meta-analysis study in which FRN and corresponding mental processes are rather spared in psychosis^44^. On the other hand, compared to CTRL group, SZ group displayed a reduction of FRN ampultude in the two emotional conditions but no difference was found between angry and neutral conditions. Unlike their healthy controls, emotional faces had less impact on choice behaviors and FRN in SZ, suggesting that their decision making is less flexible in emotional conditions. Furthermore, in contrast to the marginal correlation between the P1 and P3 scores of the PANSS and patients’ choice consistency in Li et al.^9^, significant correlations between the positive factor of PANSS and all the three measurements of reward processing (i.e., learning rate, choice consistency, and FRN) in the angry condition were reported in the current study. On one hand, it is probable that the five-factor model proposed by Wallwork et al.^45^ has more precise categorization of psychopathology than the original three-dimension model dose (Kay et al.^10^). On the other hand, our current findings support the importance of affective disturbance in the regulation of decision-making and imply a dysregulated affective arousal in the regulation of reward-based decision making in SZ. Our correlation data further indicated that both affective disturbance and alteration of FRN in the angry condition are negatively correlated with the cognitive factor of PANSS. Thus, these findings suggest that dysregulated perception of emotional faces (especially angry faces) plays an important role in the regulation of decision-making and other social-cognitive functions in patients with schizophrenia.

In addition, our correlation data also revealed that these impairments observed in our patients were mainly correlated with positive PANSS factors. These findings are somewhat in line with the predictive coding account of psychosis^46,47^, which posits that our brain constantly makes inferences on the current state of the environment by integrating prior experience with sensory inputs. In a similar vein, the discrepancy between two sources of information constitutes prediction error, which drives the learning process and updates the internal representation of the environment, similar to the role of the RPE signal in our probabilistic gambling task. It has been suggested that optimization of the inference process depends on the balance between the precision weighting of prior experience and sensory input, which are implemented by glutamatergic N-methyl-d-aspartate receptor (NMDAR) and dopaminergic signaling, respectively^48,49^. In SZ, due to the hypofunction of NMDARs and increased dopaminergic activity, this inference process is biased towards the sensory input and away from the prior belief, resulting in an abnormally strong weighting of prediction error, which, in our case, was characterized by a strong trend of increased learning rate in the angry condition. This excessively weighted prediction error would then lead to failure in sensory attenuation (aberrant salience) and overreaction to environmental change, which was characterized by lower choice consistency in our study. Interestingly, the impairment of reward processing might also be related to deficits in social functioning. Izuma, Saito, and Sadato^50^ proposed the hypothesis of common neural currency—the shared neural basis between monetary and social decision making. Levy and Glimcher^51^ conducted a meta-analysis on thirteen fMRI studies and showed that the overlapping brain areas associated with both monetary and social rewards are subregions of the ventromedial prefrontal cortex and orbitofrontal frontal cortex, validating the existence of a common value path. These studies imply a relation between deficits in social functioning and dysregulated reward processing. It is worth further investigating the neural circuits and underlying mechanism in future studies, especially in patients with schizophrenia and other psychiatric disorders.

It is worth noting that there are two major limitations in drawing inference from our findings. First, patients were not stratified on the basis of their medication which may have influence on their arousal states as well as the corresponding EEG measures^52,53^. It is therefore possible to contribute to the failure of detecting significant and reliable deficits of affective and reward processing in these patients. Second, it is possible that patients may have impaired emotion awareness that curbed their self-report of affective experience. In the present study, PANAS scores were used as a manipulation check for the influence of facial prime on participants’ subjective affective experience, and their affective arousal was objectively indexed by the P200 ERP component. For the PANAS scores, we observed that the SZ showed significant difference between prime conditions only in positive ratings, but they were not different from the CTRL (Fig. 1A). By contrast, for the P200, we identified a simple main effect of group in angry condition, where the CTRL had a significantly larger P200 than the SZ. The discrepancy between the two measurements might link to the idea that, in addition to the disturbed perception of others’ emotion, SZ also demonstrate impairment in the awareness of their own feelings^54–56^. Kimhy et al. used the Toronto Alexithymia Scale (TAS-20^57^) to assess emotion awareness in SZ^54–56^ and clinical high risk individuals^55^. They reported that there were significant differences between healthy controls and these two clinical groups in their ability to identify and describe feelings. Further, the impaired emotion awareness impacted the use of emotion regulation in patients^56^ and could account for a substantial amount of their social functioning variance^54–56^. Thus, together with our study, these findings highlight the importance of assessing participants’ level of emotion awareness while measuring their affective state with self-report questionnaires. Meanwhile, biometrics like EEG/ERP and electrocardiogram could serve as complementary measures which provide objective information regarding individual’s affective arousal.

## Method

### Participants

Thirty-eight patients who met the DSM-5 diagnostic criteria for SZ and 26 CTRL participated in this study. SZ were recruited from the outpatient clinics of the Department of Psychiatry in National Taiwan University Hospital (NTUH). Participants’ demographic data are presented in Table 2. No significant difference between SZ and CTRL was found in age and gender, except level of education (*p* < 0.001), which is consistent with previous studies with Taiwanese populations^9,58^. All participants gave fully informed written consent to participate, and all the procedures of the study followed ethical guidelines and were approved by the Research Ethics Committee of NTUH. Main exclusion criteria included (1) major neurological diseases, (2) history of serious head injury or loss of consciousness, (3) history of mental retardation or developmental disability, (4) substance abuse, and (5) menstrual period or pregnancy for females. Additional exclusion criteria for patients with schizophrenia included (1) inpatient hospitalization, (2) change in antipsychotic medication in the 3 months prior to enrollment, (3) significant extrapyramidal symptoms, and (4) comorbidity of mood disorders. Healthy controls that have family history of psychotic history among first-degree relatives were also excluded. All participants received a semistructured Diagnostic Interview for Genetic Study (DIGS) Chinese Version^59^. Psychopathology of schizophrenia was rated on the PANSS by experienced clinicians using clinical interview^10^. The average number of days between the participants’ last experimental session and their PANSS ratings is 41.13 (±19.90). Symptom subscores were derived and modified mainly based on a five-factor model proposed by Wallwork, Fortgang, Hashimoto, Weinberger, and Dickinson^45^, including positive, negative, cognitive, excitement, and depressive factors, as indicated in the middle panel of Table 2. All participants were instructed to have at least 8 h sleep at the nights before each session of experiment, and their mean reported sleeping time was 8.74 (±1.48) hours. One of the participants in this study was a smoker (averaged daily nicotine intake: 2 mg). He was instructed to abstain from smoking on each testing day of experiment to eliminate possible impact on EEG^60^.

**Table 2:** Demographic information of the patients with schizophrenia (SZ) and the healthy control (CTRL) groups.

	SZ (*N* = 38)		CTRL (*N* = 26)	
	Mean	(SD)	Mean	(SD)
Age	33.74	(7.76)	34.63	(8.59)
Gender (M:F)	20: 18		14: 12	
Education (year)	14.45	(2.31)	16.69	(1.93)
Age of onset	22.65	(5.12)	**–**	
Symptom (PANSS score)				
Total	60.37	(15.55)	–	
Positive (P1 + P3 + P5 + G9))	8.39	(3.53)	–	
Negative (N1 + N2 + N3 + N4 + N6 + G7)	12.68	(4.03)	–	
Cognitive (P2 + N5 + G11)	6.68	(2.52)	–	
Excitement (P4 + P7 + G8 + G14)	5.45	(2.37)	–	
Depressive (G2 + G3 + G6)	4.95	(1.89)	–	
Medication (*N*)				
Typical	3		–	
Atypical	24		–	
Combined	7		–	
None	4		–	

### General procedures

**Fig. 3:**
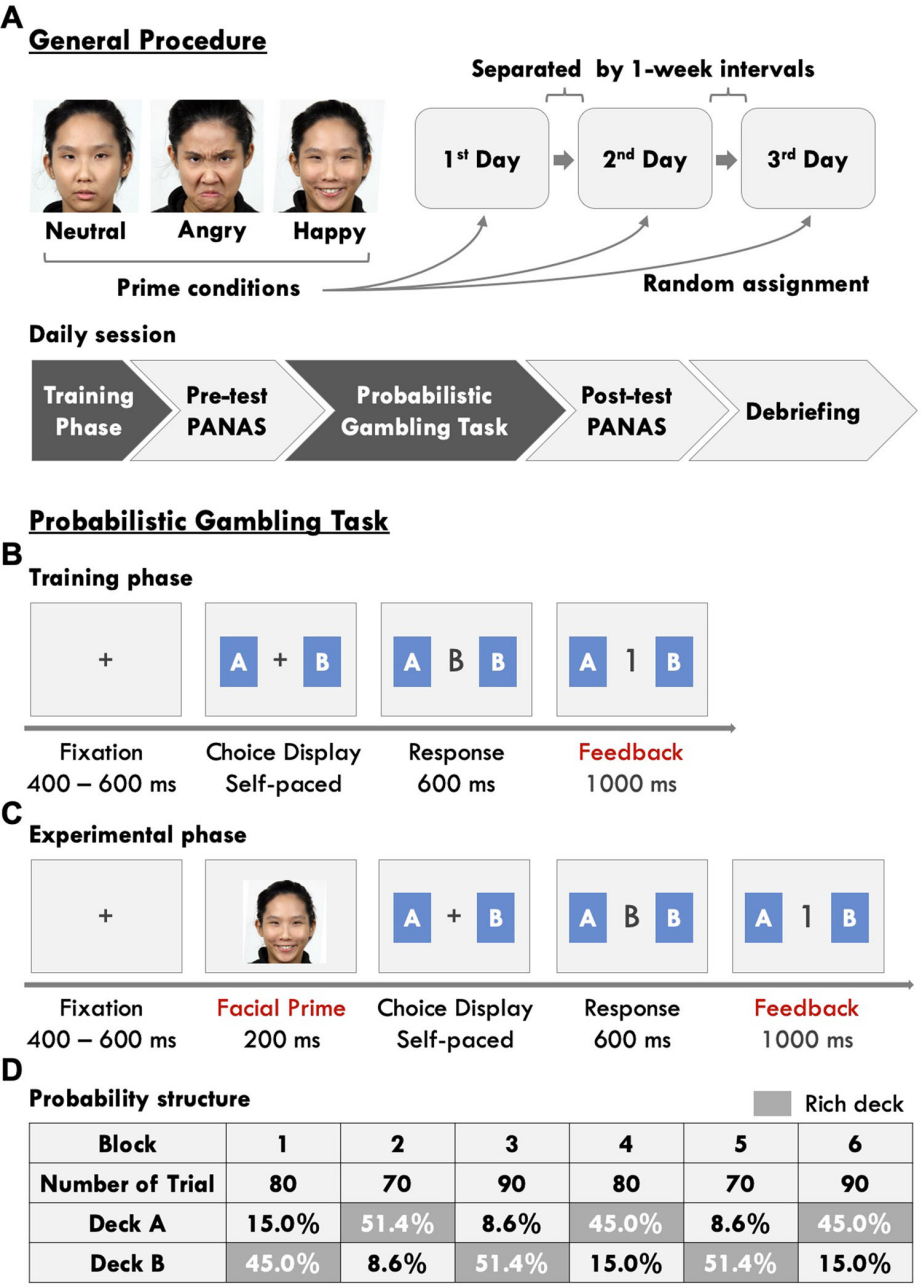
General procedure and task structure. **A** Each session was assigned one of the three kinds of facial prime conditions (i.e., neutral, happy, and angry) and comprised of five phases. **B**, **C** The training phase comprised a minimum of 20 training trials without the facial prime, whereas the experimental phase comprised 480 trials with a facial prime prior to the choice display in each trial. **D** An example of a block sequence and the underlying reward-probability structure. Reward ratios of the deck-pair varied from block to block.

As illustrated in Fig. 3A, all participants underwent three experimental sessions separated by 1 week intervals, and each session would be pseudorandomly assigned with one of the three facial prime conditions (i.e., neutral, angry, and happy conditions). The experimenter was blind to the participants’ intervention status. In each daily session, participants first engaged in the training phase of the probabilistic gambling task and then proceeded to the experimental phase after they were familiar with the rule (Fig. 3B, C). They were asked to complete the Chinese version of the Positive and Negative Affect Schedule (PANAS)^61^ before and after the experimental phase. Mean difference scores of the positive and the negative items between the post-test and pretest PANAS ratings were calculated to indicate participants’ subjective change in positive and negative affective activities, respectively. At the end of each session, participants were asked to report whether they used different strategies from previous sessions and their self-evaluation of task performance in the debriefing phase.

### Task structure in each session and behavioral data

The probabilistic gambling task employed a trial-by-trial two-choice scenario was modified from Rutledge et al.^62^ and Liu et al.^26^, which consisted of a training phase and an experimental phase. As illustrated in Fig. 3B, a pair of decks with a certain reward probability ratio (6:1 or 1:6, a total of 60%) was presented on the screen in each trial of training phase, and the participants had to choose between the two decks by key pressing. When a reward had been scheduled to their choice, a digit “1” would then be displayed on the center of the screen to represent a one-point gain. Otherwise, the displayed digit was “0”, which represented a non-reward outcome. After they completed 20 trials, the participants were asked to identify the rich and poor decks (i.e., higher vs. lower reward probability) in the pair, and the training phase was repeated until their answers were correct. In the experimental phase (Fig. 3C), participants were informed that there was a task-irrelevant face picture prior to the choice display, and that the reward distribution would be randomly shifted after certain trials. Unbeknown to the participants, the experimental phase comprised 480 trials that were pseudorandomly divided into six 70–90 trial blocks with four reward probability ratios within each deck pair (Fig. 3D). The neutral, happy, and angry prime conditions are composed of corresponding facial expression pictures selected from a culture-dependent database^63,64^. To be noted, these facial expression pictures from the database were not from any patient. The subject was instructed to maximize the total points, and a monetary reward was provided to him/her based on his/her total game scores at the end of each daily session.

### EEG acquisition and analysis

Online data were acquired through the NuAmps system (NeuroScan, El Paso, Texas, USA) from 32 scalp electrodes (QuikCap, NeuroScan, El Paso, Texas, USA) (sampling rate: 1000 Hz; online bandpass: D.C. −100 Hz; reference: nose-tip). Impedances were kept below 5 kΩ. The raw continuous data were offline preprocessed using the EDIT module from Scan 4.5 (NeuroScan, Charlotte, North Carolina, USA). In the preprocessing pipeline, the raw continuous data were subjected to a 0.1–40 Hz bandpass filter and an EOG artifact reduction procedure, by which the continuous data were mathematically corrected for eyeblink artifacts through a built-in pattern recognition algorithm^65^. The corrected continuous data were then rereferenced to the averaged mastoids and segmented into epochs of −100 to 500 ms following the onset of the facial primes to extract the ERP indices for structural encoding and the corresponding affective response to facial features (i.e., N170 and P200, respectively)^20–24,34,35^ and epochs of −100 to 900 ms following the onset of feedback display to extract ERPs for RPE signaling during reward-based decision making (i.e., raw and difference waves of the FRN)^30–33^. Baseline corrections were applied to the epoched data with respect to the mean activity of the prestimulus window. The epochs were then subjected to an artifact rejection procedure in which the epochs that contained activities exceeding ±50 µV were excluded from further analysis. The ERPs were obtained by averaging all the artifact-free epochs for each electrode and condition. For statistical analysis, the amplitude of the N170 following the facial prime was evaluated as the mean activity within the 150–210 ms poststimulus interval at channels P7 and P8; the P200 amplitude was evaluated as the mean activity within the 170–230 ms poststimulus interval at channels Fz, Cz, and Pz^20–24^.

The FRN component was considered an ERP signature of reward processing and, importantly, RPE signaling in the present study. Existing literature has identified an interaction between the factors of reward valence (i.e., win vs. loss) and reward probability (expected vs. unexpected) in the ERP associated with reward processing, and the FRN made by the different activities between unexpected loss and unexpected win is more closely related to the RPE signaling^33^. Accordingly, two raw waveforms of the FRN component were extracted for each participant. They are (1) the unexpected win, which was obtained from the ERPs in the unexpected reward-delivery trials in which the participants selected cards from the poor deck and obtained game points; and (2) the unexpected loss, which was obtained from the ERPs in the unexpected reward-omission trials in which the participants selected cards from the rich deck and obtained zero points. Then, to isolate the activities associated with the RPE signals from the other temporospatially overlapping ERP components, we subtracted the unexpected Win waveform from the unexpected loss waveform to create the FRN component (i.e., the unexpected loss – the unexpected Win difference wave)^31,33^. For statistical analysis, the amplitude of the FRN component was evaluated as the mean activity within the 300–600 ms poststimulus interval at the Fz, FCz, and Cz channels^26,29^.

### Analysis of win-stay/lose-shift behavioral strategies

To assess whether the participants could formulate adaptive strategies from the received feedbacks, we first quantified their trial-by-trial choice behavior following immediate wins and losses, with win-stay referring to the proportion of times the same deck was chosen immediately after a reward was received, and lose-shift as the proportion of times a different deck was chosen after receiving a non-reward outcome. Then, we considered the validness of feedback and separated participants’ win-stay/lose-shift behavior into (1) win-stay in rich decks, (2) win-stay in poor decks, (3) lose-shift in rich decks, (4) lose-shift in poor decks. Thus, the participants would exhibit more frequent win-stay and less frequent lose-shift to the rich decks and show exactly opposite pattern of strategy to the poor decks, if they successfully tracked the dynamic of reward distribution and optimize their choice accordingly.

### Reinforcement learning model and parameter estimation protocol

To explore the mechanism governing RPE-driven choice behavior, we fit a simplified reinforcement learning model^66,67^ closely follows the one described in Liu et al.^26^ and Li et al.^9^ to the trial-by-trial choice data from all participants. This model comprises two parts: The first part concerns how the expectation of each choice option is affected and updated by the RPE, and the second part concerns how the updated information is transformed into choice behavior. Importantly, the essence of the first part is characterized by a “learning rate (*α*)” parameter showing how rapidly the expectation is updated, and the essence of the second part is characterized by a “choice consistency (*β*)” parameter showing the choice tendency guided by the updated expectation. The model equation and the parameter estimation protocol are briefly described as follows.

First, following Sutton and Barto^67^, we used a simplified temporal difference model to characterize the dynamics of RPE signaling during the probabilistic gambling task. Specifically, the expectation of the deck was updated based on the so-called “delta learning rule”^68^. We use deck A (see Fig. 3B, C) as an example. The rule postulates that the expectation *Q*_A_(*t*) is updated as follows:

1 $$Q_{\mathrm{A}}\left( t \right) = Q_{\mathrm{A}}\left( {t-1} \right) + \alpha \cdot \left[ {R_{\mathrm{A}}\left( t \right) - Q_{\mathrm{A}}\left( {t - 1} \right)} \right],$$

where *R*_A_(*t*) is the actual outcome from choosing deck A in trial *t*, and $[R_{\mathrm{A}}\left( t \right) - Q_{\mathrm{A}}\left( {t - 1} \right)]$ is the RPE representing the discrepancy between the actual outcome received in the current trial and the expectation of Deck A from the previous trial. The parameter *α* represents the “learning rate”; it reflects how rapidly the expectation is updated.

Second, we assumed that the updated expectation *Q*_A_(*t*) from Eq. (1) is mapped into the probability of choice through a logistic transformation. Using deck A as an example, we had that the probability of choosing A in trial *t*, *P*_A_(*t*), is

2 $$P_{\mathrm{A}}\left( t \right) = \frac{{e^{\beta \cdot Q_{\mathrm{A}}(t)}}}{{e^{\beta \cdot Q_{\mathrm{A}}(t)} + e^{\beta \cdot Q_{\mathrm{B}}(t)}}}$$

The parameter *β* represents the “choice consistency”; it reflects the choice tendency guided by the updated expectation.

**Fig. 4:**
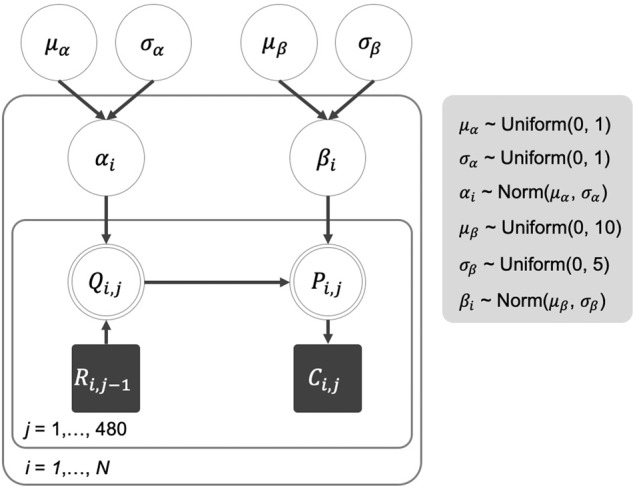
Graphic display of the Bayesian hierarchical framework for parameter estimation. Following the approach described in Lee and Wagenmakers^70^, the shaded squares, double-bordered circles, and single-bordered circles represent the observed discrete variables, deterministic continuous variables, and latent continuous variables, respectively. The arrows indicate dependencies among these variables. Specifically, *R*_i,j*–*1_ is the reward feedback (i.e., 1 or 0) received by part*i*cipant *i* in trial *j*−1, and *C*_i,j_ is the choice (i.e., A or B) made by participant *i* in trial *j*. *Q*_i,j_ and _*P*i,j_ are the expectation and choice probability of participant *i* in trial *j*.

The parameter estimation protocol also closely follows the protocol described in Liu et al.^26^. We used the Markov chain Monte Carlo (MCMC) method under the hierarchical Bayesian framework to the trial-by-trial data from the probabilistic gambling task for estimating *α* in (1) and *β* in (2). See Fig. 4 for a graphical display of the framework. For participant *i*, the learning rate parameter *α*_i_ and the choice consistency parameter *β*_I_ were each assumed to be normally distributed at the group level, with means *μ*_α_ and *μ*_β_, respectively) and standard deviations *σ*_α_ and *σ*_β_, respectively). To this end, we used WinBUGS software^69^ to perform the fit. Specifically, we used three MCMC chains, with each chain consisting of 16,000 iterations. For each chain, the first 6000 iterations were used as burn-in, and thinning with an interval of five was applied to the remaining 10,000 iterations to reduce the effect of autocorrelation. Overall, for each parameter, we obtained a total of 6000 posterior samples from the three chains. Regarding the priors for the two parameters *α* and *β*, we assumed that both *μ*_α_ and *σ*_α_ are uniformly distributed between 0 and 1, *μ*_β_ is uniformly distributed between 0 and 10 and *σ*_β_ is uniformly distributed between 0 and 5.

### Statistical analysis

Repeated measures analysis of variance was used to assess all the measures under the different combinations of conditions. Post hoc analyzes were performed using Tukey’s test when the *F*-value indicated a significant difference. Statistical significance was set as *p* < 0.05. A Greenhouse–Geisser adjustment of degrees of freedom and a Bonferroni correction were used when necessary. To evaluate the relations between indices of affective response (i.e., P200) and reward processing (i.e., FRN, learning rate, and choice consistency) and psychopathology, Pearson correlation coefficients were calculated and then corrected with a Holm–Bonferroni sequential correction to control for the impact of multiple comparisons.

## Data Availability

The data that support the findings of this study are available from the corresponding author (W.S.L.) upon reasonable request.
